# Mild hyperbaric oxygen enhanced the regeneration of the tibialis anterior muscle after the cardiotoxin-induced muscle injury

**DOI:** 10.1016/j.bbrep.2026.102638

**Published:** 2026-05-23

**Authors:** Ai Takemura, Tatsuro Egawa, Ryo Takagi, Haiyu Zhao, Ryota Iyama, Shinichiro Suzuki, Reika Fujino, Takuya Fukunaga, Tatsuya Hayashi, Satoshi Fujita

**Affiliations:** a Ritsumeikan Global Innovation Research Organization, Ritsumeikan University, Shiga, Japan; b Graduate School of Human and Environmental Studies, Kyoto University, Kyoto, Japan; c School of Nursing and Rehabilitation Sciences, Showa Medical University, Kanagawa, Japan; d Faculty of Sport and Health Science, Ritsumeikan University, Shiga, Japan

**Keywords:** Mild hyperbaric oxygen, Cardiotoxin, Muscle injury

## Abstract

Skeletal muscle injuries are widespread in everyday life and sports. The purpose of the present study was to determine whether exposure to mild hyperbaric oxygen (MHO) after muscle injury accelerates recovery. Male 8-week-old mice were injected with 30 μL of 10 μM cardiotoxin (CTX) into the left tibialis anterior (TA) muscle and saline into the right TA muscle. The mice were then divided into a normal control (CON) and an MHO (1.3 atm with 38% oxygen) group. Four weeks after CTX injection, cross-sectional area (CSA) was higher in the CTX + MHO group than in the other groups (P < 0.05). The number of centrally nucleated fibers (%) was 11.5% lower in the CTX + MHO group than in the CTX + CON group (P < 0.05), while it was higher in the CTX groups than in the saline groups regardless of MHO exposure (P < 0.05). MHO exposure had a negative main effect on the number of Pax7-positive nuclei (P < 0.05). MHO accelerated muscle recovery, characterized by a reduced proportion of centrally nucleated fibers and increased cross-sectional area of regenerating muscle fibers.

## Highlights

• Cardiotoxin induced persistent centrally nucleated muscle fibers at 4 weeks.
• Mild hyperbaric oxygen accelerated recovery after cardiotoxin injection.
• Mild hyperbaric oxygen increased fiber CSA and reduced centrally nucleated fibers.

## 1 Introduction

Skeletal muscle injuries are widespread in everyday life and in sports. Animal models of muscle injury are broadly classified into two categories: mechanically induced injuries, including muscle overload, and chemically induced injuries, in which myotoxic substances are injected into the muscle [1]. Cardiotoxin (CTX) is a typical myotoxic substance derived from *Naja pallida*, causing a temporary acute injury [2]. Some countermeasures for muscle injury, such as nutritional methods, heat and cold therapies, and pharmacotherapy, have been reported previously.

A previous study showed that hyperbaric oxygen treatment (HBOT) in an environment with 100% oxygen at 2.5 ATA accelerated satellite cell proliferation and myofiber maturation after muscle injury [3]. In muscle injury, oxygen and nutrition delivery to the muscle via vascularization is a fundamental process of regeneration [4]. In contrast to studies of a high-oxygen environment, a previous study showed that hypoxia inhibited terminal differentiation of myoblasts (both immortalized and primary) [5]. Thus, exposure to hyperbaric oxygen promoted recovery from muscle injury; however, HBOT is associated with the risk of excessive oxidative stress [6,7] or middle ear barotrauma [8]. On the other hand, mild hyperbaric oxygen (MHO), an environment at 1.25-1.30 atmospheric pressure with 36-40% oxygen, has been shown to increase oxidative metabolism without the risks of increased oxidative stress or middle ear barotrauma [9]. We have previously shown that MHO attenuated disuse-induced muscle atrophy and improved oxidative capacity [10,11]. However, there is currently no previous study that has investigated the effect of MHO on recovery from muscle injury.

The purpose of the present study was to determine whether exposure to MHO after muscle injury accelerates the recovery. We hypothesized that exposure to MHO would increase oxidative capacity and accelerate satellite cell maturation, concomitant with recovery after muscle injury induced by CTX.

## 2 Methods

### 2.1 Ethical approval

All experimental and animal care procedures were conducted in accordance with the Guidelines for the Care and Use of Laboratory Animals issued by the Institutional Animal Experiment Committee of Kyoto University (22-A-5).

### 2.2 Experimental animals and treatments

**Fig. 1:**
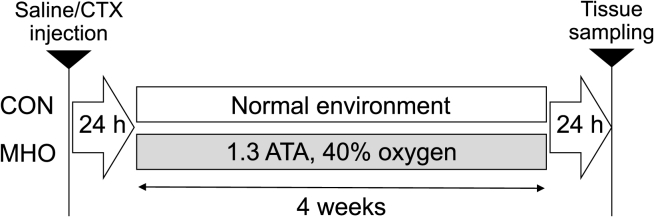
Experimental procedure. Mice were injected with CTX into the left tibialis anterior (TA) muscle and with saline into the right TA muscle. A day after injection, mice in the MHO group were exposed to MHO daily for 3 h. CTX, cardiotoxin; CON, control; MHO, mild hyperbaric oxygen; h, hour.

Male 8-week-old C57BL/6 mice were anesthetized, and 30 μl of 10 μM cardiotoxin (CTX, Latoxan, Valence, France) in saline from *Naja pallida* snake was injected into the left TA muscle (Fig. 1). Simultaneously, 30 μl of saline was injected into the right TA muscle. We randomly assigned mice to two groups: a normal control (CON, n = 10) and an MHO group (n = 10). Mice in the CON group were housed under one atmospheric pressure with 20.9% oxygen (normobaric conditions) for 4 weeks. Mice in the MHO groups were exposed to 1.3 atmospheric pressure with 38% oxygen for 3 h/day (11:30–14:30) using a chamber for 4 weeks. This time point was selected based on previous studies indicating that morphological adaptations induced by MHO are not readily detectable after short-term exposure (e.g., 2 weeks). In contrast, longer intervention periods (e.g., 4 weeks) allow reliable assessment of late-stage morphological recovery in skeletal muscle [[10], [11], [12]]. Food and water were provided ad libitum to both groups. The room was maintained in a controlled 12-h light/dark cycle (dark period from 20:00 to 08:00) at 22 °C ± 2 °C and 45–55% relative humidity. The tibialis anterior muscle was removed from each mouse under anesthesia at 24 h after the last MHO intervention, rapidly frozen in liquid nitrogen, and stored at −80 °C until further analysis.

### 2.3 Histochemical analyses

TA muscles were removed and rapidly frozen in isopentane cooled with liquid nitrogen. These muscles were mounted with optimal cutting temperature compound (Sakura Finetek, Tokyo, Japan). Transverse 10 μm-thick slices were sectioned with a cryostat at −20 °C, air-dried, and stored at −80 °C. The sections were stained with hematoxylin and eosin (H&E) and succinate dehydrogenase (SDH).

For SDH staining, tissue slides were incubated in a solution containing succinate (16 mg/mL) and nitroblue tetrazolium (1 mg/mL) in 0.2 M phosphate buffer (pH 7.5) for 15 min at 37 °C. SDH density was measured using ImageJ (NIH). Gray levels were measured at each pixel; gray level 0 was equivalent to 100% light transmission, whereas gray level 255 was equivalent to 0% light transmission.

### 2.4 Immunohistochemistry

Transverse 10 μm-thick slices kept at −80 °C. Immunohistochemistry for paired box 7 (Pax7) and *Griffonia simplicifolia* (GS)-lectin was performed as reported [3,13]. Briefly, the slides for both Pax7 and GS-lectin were fixed in 4% paraformaldehyde for 15 min at 4 °C, then washed in PBST for 15 min. After that, the Pax7 slides were fixed in methanol for 15 min at 4 °C, then washed in PBST for 15 min. After washing, the Pax7 slides were activated in 10 mM citrate buffer for 20 min at 90 °C, then washed in PBST for 15 min. After that, the slides for both Pax7 and GS-lectin were blocked with Blocking One Hist (Nakalai Tesque, Kyoto, Japan) for 1 h at RT. Then, the slides for Pax7 were incubated overnight with anti-Pax7 (mouse monoclonal antibody, DSHB, IA, USA), anti-laminin (rabbit polyclonal antibody, Sigma-Aldrich, MO, USA), Blocking One Hist, and TritonX-100 (T8787, Sigma-Aldrich) diluted 1:200, 1:500, 1:20, and 1:200 in PBST, respectively. The slides for GS-lectin were incubated overnight with anti-laminin, Blocking One Hist, and TritonX-100 diluted 1:500, 1:20, and 1:200 in PBST, respectively. The sections for Pax7 were washed for 15 min in PBST and then incubated with the secondary antibodies (goat anti-mouse IgG1-Alexa Fluor 647 and goat anti-rabbit IgG-Alexa FluorR 488; Invitrogen, MA, USA, and FUJIFILM Wako Pure Chemical Corporation, Osaka, Japan, respectively), DAPI (Dojindo Laboratories, Kumamoto, Japan), Blocking One Hist, and TritonX-100 diluted 1:250, 1: 500, 1:1,000, 1:20, and 1:200, respectively, in PBST for 1 h. The sections for GS-laminin were washed for 15 min in PBST and then incubated with the secondary antibodies (goat anti-rabbit IgG-Alexa FluorR 488), GS-lectin (labeled by Dylight594, DL-1207, Vector Laboratories, CA, USA), DAPI, Blocking One Hist, and TritonX-100 diluted 1: 500, 1:100, 1:1,000, 1:20, and 1:200, respectively, in PBST for 1 h. The sections were washed three times for 15 min in PBST. Finally, the sections were mounted in mounting solution (H-1700, Vector Laboratories). Pax7-and GS-lectin-positive cells and myonuclei were counted across the entire cross-sectional area of each TA muscle section from digitally stitched images acquired and analyzed using the BZ-X800 Analyzer (Keyence, Osaka, Japan).

### 2.5 Statistical analysis

Data are expressed as mean ± standard deviation (SD). Differences in body weight between the CON and MHO groups were evaluated using an unpaired Student's t-test. Two-way analysis of variance was performed to examine the interaction and the main effects of the leg (saline or CTX injection) and environment (CON or MHO). If significant interactions were observed, the Tukey–Kramer multiple-comparison test was performed. All statistical analyses were performed using GraphPad Prism (Ver. 9.0, Macintosh; GraphPad Software, La Jolla, CA). Statistical significance was defined as P < 0.05.

## 3 Results

### 3.1 Muscle CSA and centrally nucleated fibers

**Fig. 2:**
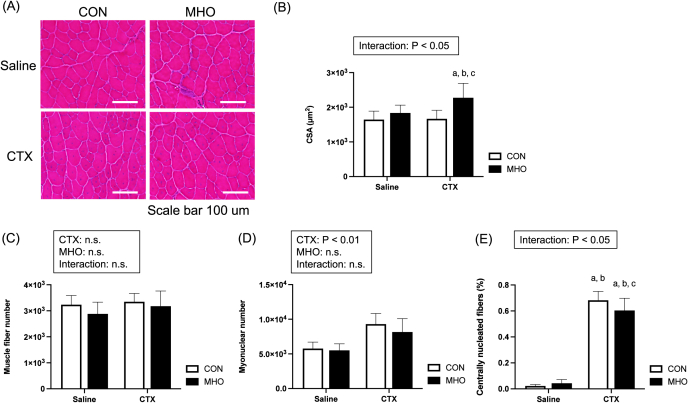
Representative images of hematoxylin and eosin (H&E) staining (A), cross-sectional area (CSA, B), muscle fiber number (C), nuclear number (D), and centrally nucleated fibers (%, E) of the tibialis anterior muscle in mice. Values are presented as mean ± standard deviation (SD) (n = 10). Statistical analysis was performed using two-way ANOVA followed by Tukey–Kramer multiple comparison test. A value of P < 0.05 was considered statistically significant. Letters a–c indicate statistically significant differences between groups (P < 0.05). Specifically, “a” indicates a significant difference compared with the Saline-CON group, “b” compared with the Saline-MHO group, and “c” compared with the CTX-CON group. CON, control; MHO, mild hyperbaric oxygen; CTX, cardiotoxin. Scale bar = 100 μm.

H&E -stained injured TA muscles at four weeks after CTX injection were observed in saline and CTX groups under the CON and MHO environment, as shown in Fig. 2A. There was an interaction of CTX and MHO on the muscle CSA. The multiple-comparison test showed that the muscle CSA was higher in the CTX leg of the MHO exposure mice than in the other three groups, i.e, saline legs in the CON and MHO groups and the CTX leg in the CON group (Fig. 2B, P < 0.05). There were no interactions and main effects in the muscle fiber number (Fig. 2C). CTX had a positive main effect on the myonuclear number (Fig. 2D, P < 0.01). There was an interaction of CTX and MHO on the number of centrally nucleated fibers relative to the number of total muscle fibers (%). The multiple-comparison test showed that the centrally nucleated fibers (%) in CTX legs was higher than saline legs regardless of environments (Fig. 2E, P < 0.0001), and it was higher in the CTX leg in the CON group than in the MHO group (Fig. 2E, P < 0.05).

### 3.2 Mitochondrial enzyme activity

**Fig. 3:**
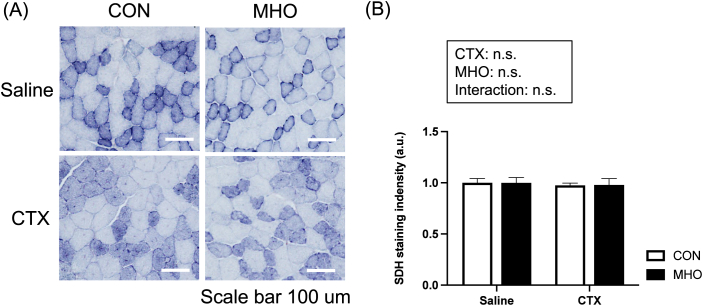
Representative images of succinate dehydrogenase (SDH) staining (A) and staining intensity (B) of the tibialis anterior muscle in mice. Values are presented as mean ± standard deviation (SD) (n = 10). Statistical analysis was performed using two-way ANOVA followed by Tukey–Kramer multiple comparison test. A value of P < 0.05 was considered statistically significant. CON, control; MHO, mild hyperbaric oxygen; CTX, cardiotoxin. Scale bar = 100 μm.

SDH-stained injured TA muscles at four weeks after CTX injection were observed in saline and CTX groups under the CON and MHO environment, as shown in Fig. 3A. There were no interactions and main effects in the SDH activity (Fig. 3B).

### 3.3 The expression of Pax7 protein

**Fig. 4:**
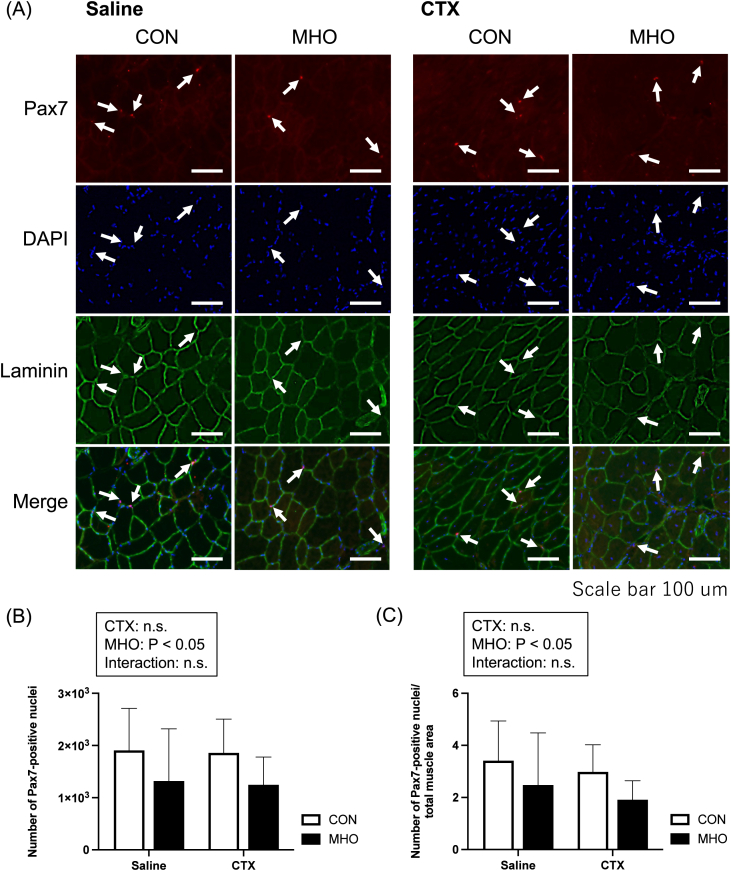
Representative images of Pax7, laminin, and 4′,6-diamidino-2-phenylindole (DAPI) immunofluorescence staining and representative merged images (A), Pax7-positive nuclei (B), and Pax7-positive nuclei relative to area (C) of the tibialis anterior muscle in mice. Values are presented as mean ± standard deviation (SD) (n = 10). Statistical analysis was performed using two-way ANOVA followed by Tukey–Kramer multiple comparison test. A value of P < 0.05 was considered statistically significant. CON, control; MHO, mild hyperbaric oxygen; CTX, cardiotoxin. Scale bar = 100 μm.

Pax7-positive nuclei were observed beside the basement membrane of injured muscles at four weeks after injury, as shown in Fig. 4A. MHO had a negative main effect on the Pax7-positive nuclei (Fig. 4B, P < 0.05) and Pax7-positive nuclei relative to total cross-sectional area of the TA muscle (Fig. 4C, P < 0.05).

### 3.4 Capillary density

**Fig. 5:**
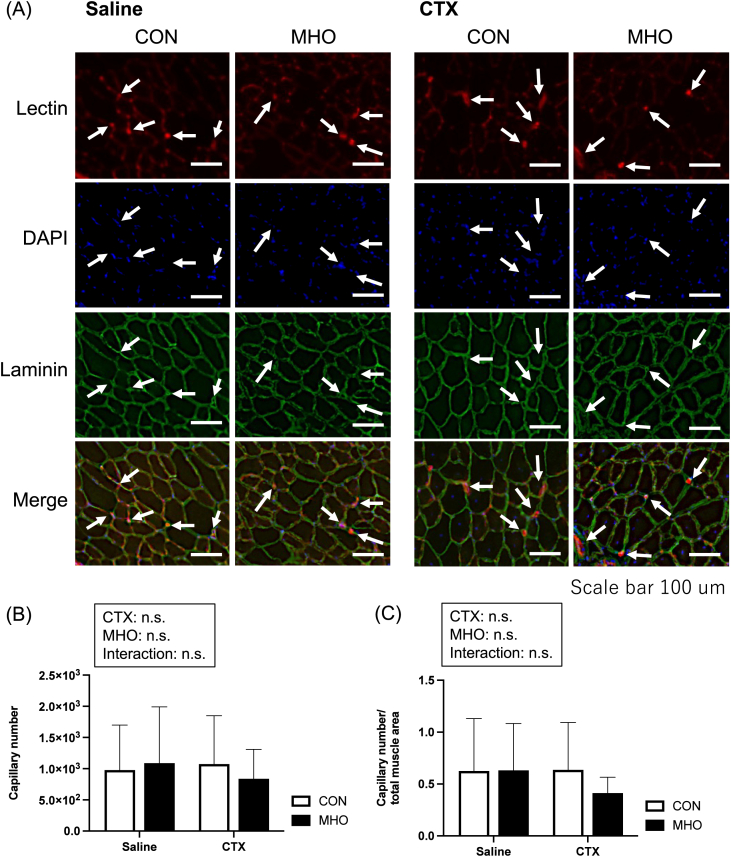
Representative images of Lectin, 4′,6-diamidino-2-phenylindole (DAPI), and laminin immunofluorescence staining and representative merged images (A), capillary number (B), and capillary number relative to area (C) of the tibialis anterior muscle in mice. Values are presented as mean ± standard deviation (SD) (n = 10). Statistical analysis was performed using two-way ANOVA followed by Tukey–Kramer multiple comparison test. A value of P < 0.05 was considered statistically significant. CON, control; MHO, mild hyperbaric oxygen; CTX, cardiotoxin. Scale bar = 100 μm.

Total capillary density (labeled by Dylight594-conjugated GS-lectin) was observed in the injured TA muscles at four weeks after CTX injection, as shown in Fig. 5A. There were no interactions and main effects on the capillary number (Fig. 5B), and their proportion relative to total cross-sectional area of the TA muscle (Fig. 5C).

## 4 Discussion

We investigated the effects of MHO on recovery after muscle injury in mice. MHO increased CSA and attenuated the number of centrally nucleated fibers after CTX injection in the mouse TA muscle.

A previous study showed that four days after CTX injection, mitochondrial damage was induced [14]. Muscle regeneration is impaired in the absence of mitochondrial remodeling, including mitochondrial dynamics [15,16]. In the present study, oxidative capacity was not affected four weeks after CTX injection. These results suggest that mitochondrial damage induced by CTX injection may be repaired during the early phase and may not persist beyond 4 weeks after injection. A previous study showed that MHO at 1.25-1.3 ATA with 36-40% oxygen enhanced muscle oxidative capacity in disuse animals [10,11]. Contrary to expectations, there was no main positive effect of MHO on SDH staining intensity in the muscle in this study. Enhanced regeneration under MHO within four weeks after CTX injection may be independent of mitochondrial enzyme activity.

In muscle injury, vascularization is a fundamental process of regeneration [4]. CTX causes myofiber myolysis by inducing depolarization of the plasma membrane [17], without affecting the vasculature or nerves [2,18]. Consistent with previous studies, no differences in muscle capillary number were observed between CTX and saline injection. Although CTX-induced injury does not directly impair the vasculature, an increase in capillary number could still potentially facilitate muscle regeneration. A previous study showed that exposure to MHO (1.25 ATA with 36% oxygen) attenuated capillary rarefaction in skeletal muscle associated with diabetes [19]. In contrast to previous research, no differences in the capillary numbers were observed between normal and MHO environments in this study. These findings suggest that enhanced regeneration in the MHO environment during the four weeks after CTX injection may not depend on vascularization.

The amount of oxygen delivered to the injured muscle area in vivo can be altered by changing atmospheric pressure, without altering capillary density. Based on Henry's Law, increased atmospheric pressure and/or oxygen concentration, as in HBOT and MHO, can increase the dissolved oxygen content in blood plasma [20,21]. After two to eight days of HBOT (2.5 ATA, 100% oxygen, 25min, 3 cycles), C2C12 myoblast growth rate and myogenin and actin production were increased [22]. On the contrary, hypoxia (0.5% oxygen) inhibited terminal differentiation of C2C12 myoblasts [5]. Based on these findings, we hypothesized that exposure to MHO would increase oxygen supply and thereby promote recovery after muscle injury by CTX. A previous study showed that HBOT (100% oxygen at 2.5 ATA) increased CSA and accelerated satellite cell proliferation and myofiber maturation 15 days after muscle injury [3]. In a mouse model of contusion injury, muscle strength was enhanced after HBOT compared with the non-intervention group [22]. In this study, similar muscle adaptations were observed as in the previous study [3], such as enhanced recovery from CTX-induced muscle injury four weeks after injection under MHO. While HBOT is associated with the risk of excessive oxidative stress [6,7] or middle ear barotrauma [8], MHO is associated with fewer side effects, including lower risks of excessive oxidative stress or middle ear barotrauma [9,21]. These findings suggest that MHO can enhance the recovery after CTX injection while minimizing the adverse effects commonly associated with HBOT.

Satellite cells are responsible for muscle regeneration after injury. These cells, in the quiescent state, express Pax7 and are activated in response to muscle injury, subsequently co-expressing both Pax7 and myogenic differentiation factor (MyoD) [23]. Most satellite cells then proliferate, downregulate Pax7, and differentiate, followed by maturation and growth [23,24]. A previous study showed that Pax7 expression peaked five days after CTX injection and gradually decreased thereafter [3]. In the present study, the number of Pax7-positive nuclei did not differ between the saline and CTX groups four weeks after injection. A previous study showed that the number of co-expressing Pax7 and MyoD cells increased three days after CTX injection and decreased eight days after injection in the HBOT (100% oxygen at 2.5 ATA) group compared with the normal environment group [3]. These results suggest that HBOT accelerated satellite cell proliferation and myofiber maturation [3]. In the current study, Pax7-positive nuclei were reduced in the MHO group 4 weeks after the treatment, regardless of saline or CTX injection. This finding may reflect either accelerated progression of satellite cell activation and differentiation or impaired maintenance of the satellite cell pool. However, because Pax7 expression is downregulated during differentiation [23] and hyperbaric oxygen treatment has been shown to enhance satellite cell proliferation and myofiber maturation in the early phase after injury [3], a detrimental effect on satellite cell maintenance appears less likely. This latter possibility, however, cannot be definitively excluded due to the lack of early time-point data. Whether this reduction reflects accelerated regeneration or altered maintenance of the satellite cell pool under MHO requires further investigation, including time-course analysis of Pax7 and MyoD expression.

In this study, CSA of injured muscle increased under an MHO environment. Consistent with the finding, a previous study showed that HBOT (100% oxygen at 2.5 ATA) increased CSA 15 days after CTX injection compared with the non-treatment group [3]. After muscle injury, inflammation and myogenesis occur within approximately the first 7 days, followed by the maturation phase [24]. A previous study reported increased muscle weight in regenerated muscle two weeks after CTX injection [25]. The increased CSA of injured muscle under MHO suggests that tissue maturation, including fiber growth, may be accelerated by MHO exposure.

The present study has a limitation. Although we evaluated the effects of MHO 4 weeks after CTX injection, this time point represents a late stage of muscle regeneration. Early time points (e.g., 3–7 days), which are critical for satellite cell activation and proliferation, were not examined. Therefore, the acute effects of MHO on satellite cell dynamics, including MyoD-associated cell proliferation, remain unclear. Future studies should consider evaluating muscle adaptation at multiple time points, including 3–7 days after CTX injection.

## 5 Conclusions

MHO under 1.3 ATA with 38% oxygen for 4 weeks accelerated muscle recovery in the TA muscle after CTX-induced injury, with a reduced proportion of centrally nucleated fibers and increased cross-sectional area of regenerating muscle fibers.

## CRediT authorship contribution statement

**Ai Takemura:** Conceptualization, Data curation, Formal analysis, Funding acquisition, Investigation, Methodology, Project administration, Resources, Software, Validation, Visualization, Writing – original draft. **Tatsuro Egawa:** Conceptualization, Data curation, Investigation, Resources, Supervision, Writing – review & editing. **Ryo Takagi:** Investigation, Methodology, Writing – review & editing. **Haiyu Zhao:** Investigation, Writing – review & editing. **Ryota Iyama:** Investigation, Writing – review & editing. **Shinichiro Suzuki:** Investigation, Writing – review & editing. **Reika Fujino:** Investigation, Writing – review & editing. **Takuya Fukunaga:** Investigation, Writing – review & editing. **Tatsuya Hayashi:** Resources, Supervision, Writing – review & editing. **Satoshi Fujita:** Conceptualization, Supervision, Writing – review & editing.

## Declaration of competing interest

The authors declare the following financial interests/personal relationships which may be considered as potential competing interests:Ai Takemura reports financial support, equipment, drugs, or supplies, statistical analysis, and writing assistance were provided by Japan Society for the Promotion of Science. If there are other authors, they declare that they have no known competing financial interests or personal relationships that could have appeared to influence the work reported in this paper.

## Data Availability

Data will be made available on request.
